# Clinical Factors Associated with Non-Obese Nonalcoholic Fatty Liver Disease Detected among US Adults in the NHANES 2017–2018

**DOI:** 10.3390/jcm11154260

**Published:** 2022-07-22

**Authors:** Zayd Adnan Razouki, Xiaotao Zhang, Jessica P. Hwang, Natalia I. Heredia

**Affiliations:** 1 Department of General Internal Medicine, The University of Texas MD Anderson Cancer Center, Houston, TX 77030, USA; zarazouki@mdanderson.org (Z.A.R.); jphwang@mdanderson.org (J.P.H.); 2 Department of Epidemiology, Division of Cancer Prevention and Population Sciences, The University of Texas MD Anderson Cancer Center, Houston, TX 77030, USA; 3 School of Public Health, The University of Texas Health Science Center at Houston, Houston, TX 77030, USA; natalia.i.heredia@uth.tmc.edu

**Keywords:** non-obese NAFLD, non-obese NAFLD fibrosis, Asian Americans, metabolic syndrome, transient elastography

## Abstract

NAFLD can occur in non-obese individuals with BMI < 25 kg/m^2^. Our goal was to examine the prevalence and clinical factors associated with non-obese NAFLD using vibration-controlled transient elastography (VCTE) with controlled attenuation parameter which estimates steatosis and fibrosis among US adults. We aggregated data from the 2017–2018 cycle of NHANES and included adults (age ≥ 20 years) with BMI < 25 kg/m^2^ with complete data for the survey, medical examination, and VCTE along with controlled attenuation parameter (CAP). We excluded participants with risks of other liver diseases. We considered patients to have non-obese NAFLD if CAP was >285 dB/m, or non-obese NAFLD fibrosis if this CAP criteria was met and liver stiffness was >8.6 kPa. We calculated the adjusted OR and 95% CI for associations with non-obese NAFLD using multivariable logistic regression. The prevalence of non-obese NAFLD was 6.2% and Asian Americans (12.2%) had the highest non-obese NAFLD prevalence. Clinical factors associated with non-obese NAFLD were advanced age and metabolic syndrome (OR_adjuste__d_ = 6.8, 95% CI 3.0–15.5). In a separate model, we found elevated glucose (OR_adjuste_ = 4.1, 95% CI 2.1–7.9), triglycerides (OR_adjuste_ = 3.8, 95% CI 1.7–8.5), and truncal fat (100-unit increase ORadjusted = 1.07, 95% CI: 1.04–1.10) were associated with higher odds of non-obese NAFLD. Meanwhile, low physical activity (OR_adjuste_ = 2.9, 95% CI 1.2–7.1) was also positively associated with non-obese NAFLD. Non-obese NAFLD is prevalent in the US and is highly associated with metabolic conditions and syndrome. Our results support the importance of considering racial/ethnic differences when investigating NAFLD in a clinical setting.

## 1. Introduction

Nonalcoholic Fatty Liver Disease (NAFLD) is a common cause of liver disease world-wide [1]. NAFLD occurs more frequently in obese individuals but can also occur in non-obese individuals who have a body mass index (BMI) < 25 kg/m^2^ [2]. The clinical significance of NAFLD in non-obese individuals remains under investigation and growing evidence suggests that non-obese NAFLD may not be a benign condition [3,4,5]. Although metabolic dysregulation in non-obese individuals appears to be less common compared to in obese individuals [6], a significant portion of these patients progress to advanced liver disease [7,8] Long-term follow-up studies suggest that patients with non-obese NAFLD can develop complications such as Type II diabetes, cardiovascular disease, and hepatocellular carcinoma at a similar rate to non-non-obese individuals, even without progression to overweight and obesity [8]. Non-obese NAFLD is an underrecognized problem in clinical practice [9,10]. Thus, understanding the prevalence of non-obese NAFLD in a US representative sample is important to inform clinicians and public health personal about the significance of non-obese NAFLD.

A previous U.S. population-based study ascertaining NAFLD using liver enzymes and ultrasound measurements estimated the prevalence of non-obese NAFLD at 7.4% [3]. Identifying NAFLD with conventional ultrasound can lead to significant inter-observer variability and limited reproducibility [11]. Vibration-controlled transient elastography (VCTE) is an accurate technique and non-invasive tool for assessing hepatic fibrosis, and the controlled attenuation parameter (CAP) score has been shown to improve standardization and quantification of hepatic steatosis [12,13]. CAP has been shown to have good inter-observer reproducibility with concordance between observers [12].

Currently, there is no U.S. population-based estimate for the prevalence of non-obese NAFLD using VCTE measurements. Thus, the principal aims of this study are to examine the prevalence and risk factors associated with non-obese NAFLD using VCTE and CAP measurements in a nationally representative sample of the U.S. population. 

## 2. Materials and Methods

### 2.1. Data Source

We conducted a cross-sectional study using aggregated data from the 2017–2018 cycle of the National Health and Nutrition Examination Survey (NHANES), a stratified, multistage probability sample representative of the civilian, non-institutionalized U.S. population. NHANES methodology and data collection have been fully described previously [14] and are available on the NHANES website (http://www.cdc.gov/nchs/nhanes.htm, accessed on 20 June 2022). In brief, participants completed a survey capturing demographic, socioeconomic, dietary, and health-related information and had a medical exam including anthropometric measurements and laboratory assessments. The National Center for Health Statistics institutional review board approved the overall NHANES, and all participants provided written consent. The University of Texas MD Anderson Cancer Center Institutional Review Board approved this specific study.

### 2.2. Study Population

**Figure 1:**
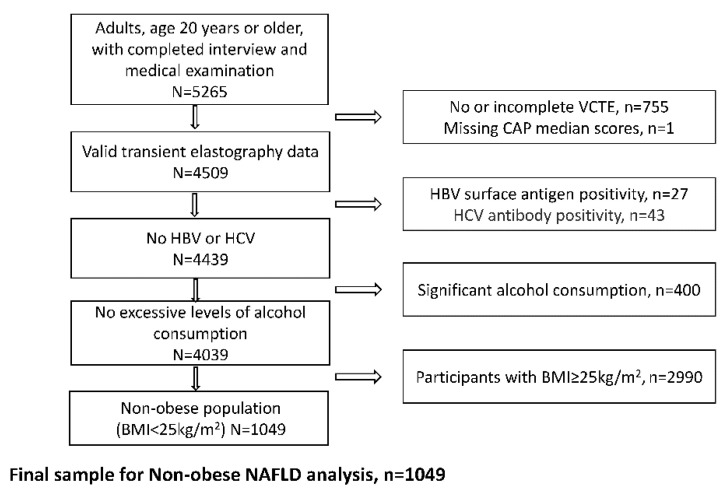
Study population flow chart.

A total of 5265 adults (age ≥ 20 years) participated in the 2017–2018 NHANES cycle and completed both the survey and medical examination. We excluded participants who did not undergo VCTE or had incomplete VCTE data (n = 755) or missing CAP scores (n = 1). We also excluded participants with risk factors for other liver diseases: chronic hepatitis B (positive hepatitis B surface antigen test, n = 27), hepatitis C exposure (positive hepatitis C antibody test, n = 43), or significant alcohol consumption (>21 drinks/week in men and >14 drinks/week in women, n = 400). Finally, we excluded participants with BMIs ≥ 25 kg/m^2^, n = 2990). The final analysis sample included 1049 participants (Figure 1).

### 2.3. NAFLD and Fibrosis Definitions

Non-obese NAFLD and non-obese NAFLD fibrosis were assessed using data obtained by VCTE with controlled attenuation. The VCTE measurements were obtained in the NHANES Mobile Examination Center, using the FibroScan^®^ model 502 V2 Touch equipped with a medium (M) or extra-large (XL) wand (probe). NHANES technicians completed a 2-day training program with the equipment manufacturer, who also certified the technicians after completing 3 satisfactory exams (Echosens^TM^ North America). For all examinations, the M probe was applied first; however, the operator switched to the XL probe if needed based on the recommendations of the device and the manufacturer’s instructions (M probe: Liver is ≤25 mm below skin; XL probe: liver is >25 mm below skin). In our final selected participants, M probe were applied for 97% of them. The operator obtained a minimum of 10 measurements from each participant, and the device calculated the median CAP and liver stiffness measurements (LSM) values along with the interquartile range (IQR). All studies were read over by a trained NHANES health technician to ensure quality. Exams were considered complete if participants fasted at least 3 **h** prior to the exam, there were 10 or more complete LSM, and the liver stiffness IQR/median < 30% [15]. The detailed procedures are described in the Liver Ultrasound Transient Elastography Procedures Manual [16]. VCTE derives LSM from the velocity of liver tissue micro-displacements induced by propagated shear waves. LSM measurements range from 1.5 kPa to 75 kPa, with higher values indicating more severe fibrosis. Simultaneously, VCTE measures the CAP value, which reflects the ultrasonic attenuation in the liver. CAP values range from 100 to 400 dB/m, with higher values indicating higher amounts of liver fat. We considered patients to have non-obese NAFLD if a CAP score ≥ 285 dB/m and to have significant non-obese NAFLD fibrosis if this CAP criteria was met along with a liver stiffness > 8.6 kPa [13].

### 2.4. Interview and Biochemistry

The interview obtained information on age, sex, race/ethnicity, marital status, household income, acculturation, smoking status, and alcohol drinking status. Alcohol drinking status was categorized as: never, light to moderate (≤2 drinks/day for men and ≤1 drink/day for women), and heavy (>2 drinks/day for men and >1 drink/day for women). Acculturation was categorized as follows: born in the U.S., lived <20, or ≥20 years in the U.S. Physical activity was collected with the Global Physical Activity Questionnaire (GPAQ) developed by the World Health Organization [17]. Adequate physical activity was defined as meeting the Physical Activity Guidelines for Americans, that is, engaging in at least 150 min a week of moderate-intensity or 75 min a week of vigorous-intensity aerobic physical activity or an equivalent combination of moderate- and vigorous-intensity aerobic physical activity [18], while inadequate physical activity was defined as anything less than meeting these guidelines. We estimated energy intake and other food components using data collected as a part of the Dietary Recall Interview that assessed the food and beverage consumed by the participants during a 24-h period before the interview. When two dietary recalls were available (n = 852, approximately 86% of our sample), assessments were averaged. Otherwise, data from one recall were used (n = 141; approximately 14% of our sample). We extracted the total fat, total percent fat, and trunk fat from the Dual-Energy X-ray Absorptiometry (DEXA), which is the most widely accepted method of measuring body composition [19,20]. Laboratory methods for measurements of Ferritin, ALT, and AST were reported in detail elsewhere [21].

### 2.5. Metabolic Factors and Comorbidities

Trained staff measured participants’ weight and height, as well as waist circumference. We calculated BMI as weight divided by height squared (kg/m^2^). Diabetes was categorized as: normal (HgbA1C < 5.7% and no self-report diabetes), pre-diabetes (HgbA1C 5.7–6.4% and no self-report diabetes), and diabetes (HgbA1C ≥ 6.5% or self-report diabetes). A homeostasis of model assessment score (HOMA) was calculated using the equation: fasting glucose (mg/dL)  ×  fasting insulin (uU/mL)/22.5 [22]. The diagnosis of metabolic syndrome required the presence of three of the following five measures, which were used to create a binary variable (with or without metabolic syndrome) according to the Adult Treatment Panel III criteria [23]: (1) waist circumference > 102 cm in men and >88 cm in women, (2) systolic blood pressure (BP) ≥ 130 mmHg or diastolic BP ≥ 85 mmHg, (3) triglycerides ≥ 150 mg/dL, (4) HDL ≤ 40 mg/dL in men or ≤50 mg/dL in women, (5) fasting glucose levels ≥ 110 mg per dL [23].

### 2.6. Statistical Analysis

Descriptive statistics were used to summarize data. We calculated non-obese NAFLD and non-obese NAFLD fibrosis prevalence among participants who had non-obese NAFLD by CAP. For between group comparisons, we used two sample *t*-test or Wilcoxon rank-sum test for continuous variables and Chi-Square test or Fisher’s exact test for categorical variables. Variables selected for assessment were determined a priori based on clinical variables expected to be associated with non-obese NAFLD. We used univariate and multivariate logistic regression models to assess factors associated with non-obese NAFLD. Backward elimination was used to build the final model, with criteria to stay *p* < 0.15. To avoid the collinearity, we conducted two separate multivariable models, with the same covariates: one included metabolic syndrome without metabolic syndrome components and the other included metabolic syndrome components but without metabolic syndrome. In addition, in our metabolic syndrome components model, we substituted waist circumference with trunk fat to evaluate the association between trunk fat with non-obese NAFLD. Since the recommended BMI cut-off points for Asians for defining overweight (23–25 kg/m^2^) and obesity (>25 kg/m^2^) are lower than those of Western populations [24]. We also conducted a sensitivity analysis that restricted the non-obese Asian Americans on BMI < 23 kg/m^2^ for non-obese NAFLD [25].

Weighted analyses were conducted using survey weights, which is fundamental to NHANES data. These weights were used to account for the complex survey design, survey non-response, post-stratification, and oversampling. By weighting, the sample becomes representative of the U.S. non-institutionalized population [26]. We used SAS 9.4 (SAS Institute INC, Cary, NC, USA) for data analyses, and *p* < 0.05 was used for statistical significance.

## 3. Results

### 3.1. Study Population

The overall study population had a mean age of 45.1 years, 41% were male, and 66% were non-Hispanic white, 10% were non-Hispanic Black, 10% were Hispanic, and 9% were Asians. Overall, 19.6% had pre-diabetes or diabetes, mean of HOMA score is 1.87 (SE = 0.1), 5.2% had metabolic syndrome, and 30.0% of participants reported inadequate physical activity. Other study population characteristics are shown in Table 1.

### 3.2. Prevalence of NAFLD and Significant Non-Obese NAFLD Fibrosis (VCTE LSM)

In total, prevalence of non-obese NAFLD by CAP was 6.2% (95% CI 3.1–9.4%), corresponding to 3.1 million U.S. adults over 20 years of age. When stratified by sex and race/ethnicity, males (7.7%) and Asian Americans (12.2%) had higher non-obese NAFLD prevalence compared with females (5.2%) and other races/ethnicities (non-Hispanic white: 6.2%; Hispanic adults: 4.4%; non-Hispanic Black: 3.8%;). The prevalence was highest in males aged 60–69 years (17.8%) and females aged 70–79 years (15.7%) (Supplemental Table S1). The prevalence of non-obese NAFLD defined by elevated liver enzymes was 7.2%. Among those with NAFLD, the prevalence of significant fibrosis (F3–F4) by VCTE LSM was 3.7% (95% CI: 0.0–7.7%) (Table 2). In a sensitivity analysis where we restricted the non-obese Asian Americans to BMI < 23 kg/m^2^, there was no statistical difference in the prevalence of non-obese NAFLD or non-obese NAFLD between the results from the original analysis vs. the sensitive analysis. The prevalence of non-obese NAFLD was 5.8%, and non-obese NAFLD fibrosis was 3.9% (Table 2). Asian American still had the highest prevalence of non-obese NAFLD (8.2%), followed by the non-Hispanic Whites (6.2%) and Hispanics (4.4%) and non-Hispanic Blacks (3.8%) (Supplemental Table S2).

### 3.3. Factors Associated with NAFLD

Table 3 shows the factors associated with non-obese NAFLD by CAP in univariate and multivariable analysis. In the multivariable analysis, advanced age was associated with non-obese NAFLD. Those with metabolic syndrome (OR_adjusted_ = 6.8, 95% CI: 3.0–15.5) and inadequate physical activity (1 unit increase OR_adjusted_ = 2.9, 95% CI: 1.2–7.1) had higher odds for non-obese NAFLD. In a separate multivariable model with individual metabolic conditions in lieu of metabolic syndrome, elevated fasting glucose (OR_adjusted_ = 4.1, 95% CI: 2.1–7.9) and elevated triglycerides (OR_adjusted_ = 3.8, 95% CI: 1.7–8.5) were independently associated with higher odds for non-obese NAFLD. When substituting waist circumference with trunk fat, trunk fat was independently associated with non-obese NAFLD (100-unit increase OR_adjusted_ = 1.07, 95% CI: 1.04–1.10) **(**Supplementary Table S3). In the sensitivity analysis that restricted the BMI < 23 kg/m^2^ for Asian Americans, similar risk factors were identified (Supplemental Table S4).

## 4. Discussion

In a nationwide population-based study, the prevalence of non-obese NAFLD using VCTE CAP measurement was 6.2%. Non-obese NAFLD was independently associated with advanced age, metabolic syndrome, and certain components of metabolic syndrome including high triglycerides and fasting blood glucose levels, but not associated with other components, including low HDL levels, high blood pressure, and elevated waist circumference. Non-obese NAFLD was also associated with trunk fat, inadequate physical activity levels, and current smoking status.

The prevalence of non-obese NAFLD reported here is lower than the global prevalence estimates of two recent systemic reviews, 9.7% [27] and 10.6% [28]. A study using NHANES III data from 1988–1994 estimated the prevalence of lean NAFLD to be 7.39% ± 0.65% when defining NAFLD using ultrasound [3]. Some of the variation in the prevalence of non-obese NAFLD can be attributed to the use of various diagnostic tools, thresholds to define NAFLD, and a difference in the characteristics of study participants. Higher prevalence of fibrosis among obese and non-obese individuals was reported in a previous study published by our group [29]. Although our data might suggest that fibrosis may be less of a concern in non-obese individuals, caution should be exercised given the small number of individuals with fibrosis in our dataset. To our knowledge, we are the first US population-based study to report the prevalence of non-obese NAFLD fibrosis using VCTE.

We highlight differences in the prevalence of non-obese NAFLD among different racial/ethnic groups. Although these differences did not reach statistical significance, Asian Americans had the highest prevalence of non-obese NAFLD compared to other racial/ethnic groups, whether non-obese NAFLD was defined as BMI < 25 kg/m^2^ or BMI < 23 kg/m^2^ (12.2%, 8.2%, respectively). This finding supports previous research that found that Asian American individuals with NAFLD had lower average BMI compared to individuals from other racial/ethnic groups with NAFLD [30]. The high prevalence of non-obese NAFLD in Asian Americans is in contrast to other U.S. population findings that indicate that both obese and non-obese Hispanic adults combined have the highest prevalence of NAFLD [29]. Our results support the importance of considering racial/ethnic differences when investigating NAFLD in clinical settings.

About a quarter of those who had non-obese NAFLD met criteria for metabolic syndrome, which is considerably less (40%) when compared to those who have NAFLD in general (i.e., obese and non-obese) [29]. Metabolic syndrome was independently associated with non-obese NAFLD, a finding which aligns with smaller, non-US-based studies that used ultrasound and VCTE with CAP scores to diagnose NAFLD in non-obese individuals [31,32,33]. Our results support the notion that NAFLD in non-obese and obese individuals shares a common altered metabolic profile that can increase the risk of cardiovascular diseases [33,34]. Like our study, non-obese NAFLD was independently associated with impaired fasting glucose [3,32,33] and high triglyceride levels [32,33]. Here, we demonstrate a unique and significant association between non-obese NAFLD and trunk fat, but not waist circumference. Waist circumference may not be an accurate proxy for trunk fat since it includes subcutaneous fat that is believed to be metabolically inert. When considering diagnosis of NAFLD in non-obese individuals, trunk fat, if available, should be considered instead of waist circumference.

Lifestyle modification including a lower caloric diet is a major pillar of NAFLD management [35,36]. Previous studies including both obese and non-obese individuals with NAFLD suggested that high intake of soft drinks and animal protein are associated with NAFLD [37], but other studies have shown null associations with these food groups [38,39]. The association of specific macronutrients in non-obese NAFLD has not been widely studied. In our study, macronutrients including high fat, carbohydrates, protein, and micronutrient including Vitamin E were not independently associated with NAFLD. However, the role of dietary intake in non-obese NAFLD may be better addressed in prospective studies. The association between inadequate physical activity and non-obese NAFLD is consistent with previous research among obese and non-obese individuals with NAFLD, in which both aerobic physical activity and resistance training exercises were associated with lower intra-hepatic triglyceride levels and/or lower risk of NAFLD [39,40,41].

Our study has several strengths. We are the first population-based study to report the prevalence of non-obese NAFLD using VCTE in the US. We also included traditional factors associated with non-obese NAFLD that are supported by a large body of prior work [3,5,8,25,27,28,30,31,33,39]. However, our study has several limitations. First, the small number of individuals with fibrosis did not allow us to confidently report an accurate estimate of non-obese NAFLD in the US population, explore factors associated with fibrosis, nor conduct stratification analysis based on the socioeconomic statuses of participants such as age, sex, and race/ethnicity. Second, the cross-sectional nature of our study did not allow us to infer causation. Finally, we did not have information on weight changes and genetic factors that have been linked to NAFLD in non-obese individuals [42,43,44]. 

## 5. Conclusions

In conclusion, the prevalence of non-obese NAFLD is 6.2% using a representative sample of US adults and VCTE with CAP measurements, and Asian Americans had the highest prevalence of non-obese NAFLD compared to other racial/ethnic groups. To help inform clinical practice and early diagnosis, we extend the knowledge about factors that are associated with non-obese NAFLD, including metabolic syndrome, high triglycerides, elevated fasting blood glucose levels, trunk fat, and physical inactivity. Further, we highlight the need for more research to identify feasible and appropriate factors to assist in detecting non-obese NAFLD in clinical practice, as well as the importance of considering racial/ethnic differences when investigating NAFLD in clinical settings.

## Figures and Tables

**Table 1:** Characteristics of factors according to non-obese NAFLD status by CAP.

Variables		Total		Non-Obese NAFLD Status				
				Yes		No		*p*-Value
				(CAP ≥ 285 dB/m)		(CAP < 285 dB/m)		
				(n = 95)		(n = 954)		
		n	Weighted % ± SE	n	Weighted % ± SE	n	Weighted % ± SE	
Age								
	Mean ± SE	1049	45.1 ± 0.8	95	58.4 ± 2.4	954	44.2 ± 0.7	<0.0001
	20–29	236	28.6 ± 2.5	5	4.6 ± 2.1	231	30.2 ± 2.4	
	30–39	171	16.4 ± 1.7	5	9.9 ± 4.7	166	16.9 ± 1.8	
	40–49	124	12.4 ± 1.5	10	7.9 ± 3.2	114	12.7 ± 1.7	
	50–59	162	17.8 ± 2.1	19	25.3 ± 6.7	143	17.3 ± 2.2	
	60–69	174	14.0 ± 1.8	29	27.5 ± 7.6	145	13.1 ± 1.8	
	70–79	110	7.0 ± 0.9	14	16.3 ± 4.8	96	6.4 ± 0.9	
	80–89	72	3.8 ± 0.7	13	8.6 ± 4.0	59	3.5 ± 0.7	
Sex								0.17
	Male	485	41.2 ± 2.4	50	50.8 ± 6.6	435	40.6 ± 2.5	
	Female	564	58.8 ± 2.4	45	49.2 ± 6.6	519	59.4 ± 2.5	
Race								0.0492
	Non-Hispanic White	367	66.4 ± 2.7	34	66.1 ± 7.5	333	66.4 ± 2.7	
	Non-Hispanic Black	219	10.1 ± 1.3	9	6.1 ± 3.7	210	10.4 ± 1.3	
	Hispanics	149	10.4 ± 1.7	11	7.3 ± 2.4	138	10.7 ± 1.7	
	Asian Americans	260	9.4 ± 1.5	37	18.5 ± 5.5	223	8.8 ± 1.5	
	Other	54	3.6 ± 1.5	4	2.0 ± 1.0	50	3.7 ± 0.6	
Acculturation								0.004
	Born in the U.S.	689	82.3 ± 1.5	42	62.5 ± 9.3	647	83.6 ± 1.6	
	<20 years in the U.S.	161	9.0 ± 1.2	23	15.9 ± 5.7	138	8.5 ± 1.3	
	≥20 years in the U.S.	189	8.7 ± 0.8	30	21.6 ± 6.4	159	7.8 ± 0.8	
Marital status								0.0003
	Never married	246	26.6 ± 1.6	5	4.5 ± 2.1	241	28.1 ± 1.5	
	Married or living with partner	587	56.2 ± 1.7	69	73.9 ± 5.9	518	55.0 ± 2.0	
	Windowed, divorced or separated	214	17.3 ± 0.8	21	21.6 ± 5.8	193	16.9 ± 1.0	
Household income								0.84
	<$55,000	493	40.0 ± 2.8	36	41.7 ± 7.8	446	39.9 ± 3.0	
	≥$55,000	477	60.0 ± 2.8	83	58.3 ± 7.8	441	60.1 ± 3.0	
Smoking								0.045
	Nonsmoker	647	76.1 ± 2.3	60	86.7 ± 6.6	587	75.5 ± 2.4	
	Former smoker	31	3.2 ± 0.7	3	7.8 ± 6.0	28	2.9 ± 0.7	
	Current smoker	187	20.7 ± 2.2	6	5.5 ± 2.9	181	21.5 ± 2.4	
Alcohol drinking								0.002
	Never	356	25.7 ± 2.5	45	33.8 ± 5.2	311	25.1 ± 2.5	
	Light to Moderate	399	39.2 ± 2.3	38	53.1 ± 6.6	361	38.2 ± 2.6	
	Heavy	293	35.2 ± 2.1	12	13.2 ± 5.4	281	36.6 ± 2.0	
Physical activity								0.0009
	Inadequate	313	30.0 ± 2.4	41	57.6 ± 8.4	272	28.4 ± 2.5	
	Adequate	476	70.1 ± 2.4	29	42.4 ± 8.4	447	71.6 ± 2.5	
Total energy intake/day (Mean ± SE)		852	2069 ± 55	79	1841 ± 113	773	2084 ± 58	0.06
Carbohydrate intake/day (Mean ± SE)		852	245.8 ± 7.5	79	228.4 ±14.9	773	247.0 ± 8.0	0.28
Total fat intake/day (Mean ± SE)		852	82.8 ± 2.5	79	72.5 ± 5.4	773	83.5 ± 2.6	0.09
Total protein intake/day (Mean ± SE)		852	79.4 ± 2.4	79	68.4 ± 4.7	773	80.1 ± 2.4	0.02
Total fiber intake/day (Mean ± SE)		852	245.8 ± 7.5	79	17.3 ± 0.9	773	17.9 ± 0.9	0.64
Total sugar intake per day (Mean ± SE)		852	105.9 ± 4.4	79	99.6 ± 10.5	773	106.4 ± 4.7	0.56
Diabetes								<0.0001
	Normal	679	80.4 ± 1.5	32	49.8 ± 7.1	647	82.4 ± 1.3	
	Pre-diabetes	203	14.4 ±1.2	25	20.3 ± 5.6	178	14.0 ± 1.3	
	Diabetes	97	5.2 ±0.7	28	29.9 ± 8.4	69	3.6 ± 0.6	
Self-reported CVD								0.007
	Yes	84	5.7 ± 0.7	14	18.7 ± 8.4	70	4.8 ± 0.7	
	No	956	94.3 ± 0.7	80	81.3 ± 8.4	876	95.2 ± 0.7	
BMI (Mean ± SE)		1049	22.0 ± 0.1	95	23.3 ± 0.1	954	21.9 ± 0.1	<0.0001
	<23 kg/m^2^	608	59.7 ±2.6	29	24.0 ± 3.8	579	62.0 ± 2.8	
	>23 kg/m^2^	441	40.3 ± 2.6	66	76.0 ± 3.8	375	38.0 ± 2.8	
Metabolic Syndrome								<0.0001
	Yes	74	5.2 ± 0.9	29	26.0 ± 6.3	45	3.8 ± 0.6	
	No	875	94.8 ± 0.9	57	74.0 ± 6.3	818	96.2 ± 0.6	
Elevated waist circumference								0.022
	Yes	92	9.3 ± 1.7	17	20.3 ± 6.9	75	8.6 ± 1.7	
	No	942	90.7 ± 1.7	77	79.7 ± 6.9	865	91.4 ± 1.7	
Elevated triglycerides								<0.0001
	Yes	182	16.5 ± 1.5	44	48.5 ± 10.2	138	14.3 ± 1.3	
	No	800	83.5 ± 1.5	90	51.5 ± 10.2	754	85.7 ± 1.3	
Low HDL cholesterol								0.009
	Yes	141	13.7 ± 1.6	28	27.5 ± 8.0	113	12.8 ± 1.4	
	No	845	86.3 ± 1.6	62	72.5 ± 8.0	783	87.2 ± 1.4	
Elevated blood pressure								0.023
	Yes	330	24.5 ± 2.5	48	41.3 ± 7.5	282	23.3 ± 2.7	
	No	698	75.5 ± 2.4	45	58.7 ± 7.5	653	76.7 ± 2.7	
Elevated fasting glucose								<0.0001
	Yes	95	6.1 ± 1.2	31	31.5 ± 7.7	64	4.3 ± 0.7	
	No	887	93.9 ± 1.2	59	68.5 ± 7.7	828	95.7 ± 0.7	
AST (IU/L) (Mean ± SE)		978	21.4 ± 0.7	89	21.8 ± 1.0	889	21.4 ± 0.7	0.72
ALT (IU/L) (Mean ± SE)		983	18.2 ± 0.5	90	20.8 ± 0.9	893	18.0 ± 0.5	0.03
LSM value (kPa, Mean ± SE)		1049	4.7 ± 0.1	95	5.5 ± 0.7	954	4.7 ± 0.1	<0.0001
Ferritin (ng/mL) (Mean ± SE)		997	117.7 ± 7.7	90	127.4 ± 14.8	907	117.1 ± 7.4	0.34
DEXA								
	Total Fat (g)	565	17234 ± 260	33	21101 ± 901	532	17056 ± 265	0.001
	Total percent fat (%)	565	28.1 ± 0.3	33	31.7 ± 1.4	532	27.9 ± 0.3	0.02
	Trunk fat (g)	613	7405 ± 159	35	10413 ± 556	578	7271 ± 166	<0.0001
Vitamin E (mg)		852	9.9 ± 0.5	79	7.8 ± 0.7	773	10.0 ± 0.5	0.04
HOMA score		493	1.87 ± 0.1	48	4.4 ±0.9	445	1.71±0.1	0.007

Elevated waist circumference: more than 102 cm (40 in) in men and more than 88 cm (35 in) in women; elevated triglyceride levels: at least 150 mg per dL (1.70 mmol per L); low high-density lipoprotein cholesterol levels: less than 40 mg per dL (1.04 mmol per L) in men and less than 50 mg per dL (1.30 mmol per L) in women; elevated blood pressure: at least 130/85 mm Hg; and elevated fasting glucose levels: at least 110 mg per dL (6.10 mmol per L); AST: aspartate aminotransferase; ALT: alanine aminotransferase; LSM: liver stiffness measure; DEXA: Dual-Energy X-ray Absorptiometry.

**Table 2:** Prevalence of non-obese NAFLD and non-obese NAFLD fibrosis.

		N	%	95% CI
Non-obese NAFLD defined by Steatosis (CAP ≥ 285 dB/m)				
	No	954	93.8	90.6–96.9
	Yes	95	6.2	3.1–9.4
Non-obese NAFLD defined by Steatosis (CAP ≥ 285 dB/m, restricting Non-obese Asian Americans on BMI < 23 kg/m^2^)				
	No	863	94.2	90.9–97.5
	Yes	72	5.8	2.5–9.1
Non-obese NAFLD Fibrosis by VCTE LSM (Among NAFLD participants defined by CAP, LSM ≥ 8.6)				
	No	91	96.3	92.3–100
	Yes	4	3.7	0.0–7.7
Non-obese NAFLD Fibrosis by VCTE LSM (Among NAFLD participants defined by CAP, LSM ≥ 8.6, restricting Non-obese Asian Americans on BMI < 23 kg/m^2^)				
	No	69	96.1	91.5–100
	Yes	3	3.9	0–8.4

**Table 3:** Multivariable analysis for factors associated with non-obese NAFLD.

Variables		Crude OR	95% CI	Multivariable Adjusted OR ^a^	95% CI
Age					
	1 unit increase	1.05	1.02–1.07		
	20–29	Ref		Ref	
	30–39	3.9	1.1–13.7	3.1	0.9–11.1
	40–49	4.1	1.0–16.5	3.3	0.7–15.7
	50–59	9.7	2.8–32.8	5.6	1.4–21.6
	60–69	13.9	4.2–45.4	7.9	2.4–26.1
	70–79	16.9	4.9–57.9	7.9	1.6–39.5
	80–89	16.1	4.0–64.1	5.3	0.8–33.8
Sex					
	Male	1.5	0.8–2.8	2.3	0.96–5.63
	Female	Ref		Ref	
Race					
	Non-Hispanic White	Ref		Ref	
	Non-Hispanic Black	0.6	0.2–2.3	0.7	0.2–1.9
	Hispanics	0.7	0.3–1.4	0.7	0.3–1.5
	Asian Americans	2.1	0.9–5.1	1.5	0.6–3.5
	Other	0.5	0.1–2.3	0.8	0.3–2.3
Household income					
	<USD 55,000	Ref		Ref	
	≥USD 55,000	0.9	0.4–2.0	0.8	0.4–1.7
Acculturation					
	Born in the U.S.	Ref			
	<20 years in the U.S.	2.5	0.9–7.3		
	≥20 years in the U.S.	3.7	1.3–10.3		
Marital status					
	Never married	Ref			
	Married or living with partner	8.5	2.9–24.3		
	Windowed, divorced or separated	8.0	2.3–28.1		
BMI (1 unit increase)		1.6	1.4–1.9		
	BMI < 23 kg/m2	Ref			
	BMI > 23 kg/m2	5.2	3.2–8.5		
Metabolic Syndrome		8.9	4.0–19.9	6.8	3.0–15.5
	Elevated waist circumference *	2.7	1.1–6.8	2.1	0.9–5.1
	Elevated triglycerides *	5.6	2.2–14.6	3.8	1.7–8.5
	Low HDL cholesterol *	2.6	1.1–6.2	1.7	0.9–3.1
	Elevated blood pressure *	2.3	1.0–5.2	1.0	0.5–2.4
	Elevated fasting glucose *	10.1	5.3–19.3	4.1	2.1–7.9
Self-reported CVD		4.6	1.2–17.4		
Smoking					
	Nonsmoker	Ref		Ref	
	Former smoker	2.3	0.4–13.6	2.1	0.2–20.6
	Current smoker	0.2	0.1–0.7	0.2	0.1–0.7
Alcohol drinking					
	Never	Ref			
	Light to Moderate	1.0	0.6–1.9		
	Heavy	0.3	0.1–0.6		
Physical activity					
	Inadequate	3.4	1.5–7.6	2.9	1.2–7.1
	Adequate	Ref		Ref	
Macronutrients					
	Average total energy intake (1000-unit increase)	0.70	0.5–1.1		
	Average Carbohydrate intake (100-unit increase)	0.9	0.7–1.2		
	Average Total fat (100-unit increase)	0.5	0.2–1.3		
	Average Protein intake per day (1 unit increase)	0.99	0.98–1.00		
	Average fiber intake per day (1 unit increase)	0.99	0.97–1.02		
	Average total sugar intake per day (1 unit increase)	0.999	0.993–1.004		
AST (IU/L) (1 unit increase)		1.00	0.99–1.02	0.98	0.92–1.03
ALT (IU/L) (1 unit increase)		1.01	1.00–1.02	1.03	0.98–1.07
Ferritin (ng/mL) (100-unit increase)		1.05	0.95–1.16		
DEXA					
	Total Fat (g, 100-unit increase)	1.02	1.0–1.03		
	Total percent fat (%, 1 unit increase)	1.08	1.01–1.16		
	Trunk fat (g, 100-unit increase)	1.06	1.02–1.09		
Vitamin E (mg) (1 unit increase)		0.93	0.86–1.02		
HOMA score		1.43	1.02–2.01		

* Final model adjusted without metabolic syndrome. ^a^ Final model including age, sex, race, household income, physical activity, smoking status, ALT, AST, and with either metabolic syndrome or metabolic syndrome components (elevated waist circumference, elevated triglycerides, low HDL cholesterol, elevated blood pressure and elevated fasting glucose), using backward elimination methods, with stay p < 0.15. DEXA: Dual-Energy X-ray Absorptiometry; ALT: alanine aminotransferase; AST: aspartate aminotransferase; CVD: cardiovascular disease.

## Data Availability

The datasets generated and/or analyzed during the current study are not publicly available but are available from the corresponding author on reasonable request.

## Supplementary Materials

The following supporting information can be downloaded at: https://www.mdpi.com/article/10.3390/jcm11154260/s1, Table S1. Weighted prevalence of non-obese NAFLD by age group, sex, and race/ethnicity; Table S2. Prevalence of non-obese NAFLD by race/ethnicity after restricting non-obese Asians on BMI < 23 kg/m^2^; Table S3. Multivariable analysis for factors associated with non-obese NAFLD, substituting trunk fat for waist circumference; Table S4. Multivariable analysis for factors associated with non-obese NAFLD, restricting non-obese Asian on BMI < 23 kg/m^2^.
