# Supplementary material for: Clinical Factors Associated with Non-Obese Nonalcoholic Fatty Liver Disease Detected among US Adults in the NHANES 2017–2018
Source: J Clin Med. 2022 Jul 22;11(15):4260. doi: 10.3390/jcm11154260 (PMC9331553; doi:10.3390/jcm11154260)
Supplement: Supplementary file 1 [file jcm-11-04260-s001.zip › jcm-1815664-supplementary.pdf]

Table S1. Weighted prevalence of non-obese NAFLD by age group, sex and race/ethnicity

Sex by age	Non-Hispanic White (N=367)		Non-Hispanic Black (N=219)		Hispanics (N=149)		Asian Americans (N=260)		Total* (N=1049)	
	**Prevalence**	**95% CI**	**Prevalence**	**95% CI**	**Prevalence**	**95% CI**	**Prevalence**	**95% CI**	**Prevalence**	**95% CI**
**NAFLD by CAP**										
**Male**	7.3	1.4-13.2	5.4	0.0-11.3	8.2	2.3-14.0	14.8	7.0-22.6	7.7	3.7-11.7
20-29	1.0	0.0-3.3	5.5	0.0-13.9	NA	NA	2.6	0.0-4.8	1.7	0.0-3.5
30-39	NA	NA	NA	NA	14.0	0.0-45.2	7.3	0.0-21.6	2.0	0.0-5.2
40-49	10.0	0.0-26.1	11.3	0.0-32.6	NA	NA	7.2	0.0-23.3	8.4	0.0-18.4
50-59	14.0	0.0-32.0	6.5	0.0-17.9	24.8	0.0-71.8	13.7	0.0-34.6	14.0	2.0-26.0
60-69	19.9	0.0-48.1	6.7	0.0-13.7	5.4	0.0-18.0	27.1	2.9-51.3	17.8	0.0-37.9
70-79	8.6	0.0-24.2	10.1	0.0-29.7	40.4	0.0-100	33.8	11.8-55.7	13.0	1.7-24.3
80-89	8.6	0.0-18.5	NA	NA	33.6	0.0-91.8	NA	NA	13.4	1.7-24.4
**Female**	5.4	0.9-10.0	1.7	0.0-5.2	2.4	0.2-4.6	10.7	6.8-14.7	5.2	1.9-8.5
20-29	NA	NA	2.8	0.0-9.1	NA	NA	NA	NA	0.3	0.0-1.0
30-39	7.3	0.0-17.9	NA	NA	NA	NA	4.7	0.0-13.3	4.9	0.0-11.5
40-49	1.2	0.0-4.0	6.2	0.0-22.2	NA	NA	8.1	0.0-18.9	2.0	0.0-4.3
50-59	5.7	0.0-14.1	NA	NA	3.4	0.0-10.2	14.7	3.6-25.8	6.3	0.0-12.9
60-69	6.8	0.0-14.6	NA	NA	30.5	0.8-60.3	22.8	9.9-35.8	8.8	1.8-15.7
70-79	16.9	0.0-38.8	NA	NA	9.4	0.0-24.2	27.1	3.7-50.5	15.7	0.0-31.8
80-89	16.4	0.0-41.9	NA	NA	NA	NA	18.5	0.0-58.5	14.5	0.0-35.8
**Total**	6.2	1.9-10.5	3.8	0.0-8.5	4.4	2.0-6.8	12.2	8.5-16.0	6.2	3.1-9.4
**Overall NAFLD**	36.4	32.0-40.9	27.0	23.6-30.3	42.5	38.5-46.6	33.5	28.6-38.3	36.1	33.2-39.1

"NA", indicate the prevalence is 0, unable to calculate the weighted prevalence of US population

*Other Race - Including Multi-Racial group is not shown

Table S2. Prevalence of non-obese NAFLD by race/ethnicity after restricting non-obese Asians on BMI<23kg/m^2

		N	%	95% CI
**Non-Hispanic White**				
	No	333	93.8	89.5-98.1
	Yes	34	6.2	1.9-10.5
**Non-Hispanic Black**				
	No	210	96.2	91.5-100
	Yes	9	3.8	0-8.5
**Hispanics**				
	No	138	95.6	93.2-98.0
	Yes	11	4.4	2.0-6.8
**Asian**				
	No	132	91.8	89.0-94.6
	Yes	14	8.2	5.4-11.0
**Other**				
	No	50	96.7	91.9-100
	Yes	4	3.4	0-8.1

Table S3. Multivariable analysis for factors associated with non-obese NAFLD**,** substituting trunk fat for waist circumference

**Variables**	**Crude OR**	**95%CI**	**Multivariable adjusted OR[a]**	**95%CI**
**Age**				
1 unit increase	1.05	1.02-1.07		
20-29	Ref		Ref	
30-39	3.9	1.1-13.7	3.1	0.9-11.1
40-49	4.1	1.0-16.5	3.3	0.7-15.7
50-59	9.7	2.8-32.8	5.6	1.4-21.6
60-69	13.9	4.2-45.4	7.9	2.4-26.1
70-79	16.9	4.9-57.9	7.9	1.6-39.5
80-89	16.1	4.0-64.1	5.3	0.8-33.8
**Sex**				
Male	1.5	0.8-2.8	2.3	0.96-5.63
Female	Ref		Ref	
**Race**				
Non-Hispanic White	Ref		Ref	
Non-Hispanic Black	0.6	0.2-2.3	0.7	0.2-1.9
Hispanics	0.7	0.3-1.4	0.7	0.3-1.5
Asian Americans	2.1	0.9-5.1	1.5	0.6-3.5
Other	0.5	0.1-2.3	0.8	0.3-2.3
**Household income**				
<$55,000	Ref		Ref	
≥$55,000	0.9	0.4-2.0	0.8	0.4-1.7
**Acculturation**				
Born in the U.S.	Ref			
<20 years in the U.S.	2.5	0.9-7.3		
>=20 years in the U.S.	3.7	1.3-10.3		
**Marital status**				
Never married	Ref			
Married or living with partner	8.5	2.9-24.3		
Windowed, divorced or separated	8.0	2.3-28.1		
**BMI (1 unit increase)**	1.6	1.4-1.9		
BMI<23 kg/m^2	Ref			
BMI>23kg/m^2	5.2	3.2-8.5		
**Metabolic Syndrome**	8.9	4.0-19.9	6.8	3.0-15.5
**Elevated waist circumference**	2.7	1.1-6.8		
**Elevated triglycerides***	5.6	2.2-14.6	0.8	0.1-4.6
**Low HDL cholesterol***	2.6	1.1-6.2	1.1	0.5-2.5
**Elevated blood pressure***	2.3	1.0-5.2	0.7	0.3-1.4
**Elevated fasting glucose***	10.1	5.3-19.3	13.6	4.4-42.2
**Self-reported CVD**	4.6	1.2-17.4		
**Smoking**				
Nonsmoker	Ref		Ref	
Former smoker	2.3	0.4-13.6	2.1	0.2-20.6
Current smoker	0.2	0.1-0.7	0.2	0.1-0.7
**Alcohol drinking**				
Never	Ref			
Light to Moderate	1.0	0.6-1.9		
Heavy	0.3	0.1-0.6		
**Physical activity**				
Inadequate	3.4	1.5-7.6	2.9	1.2-7.1
Adequate	Ref		Ref	
**Macronutrients**				
Average total energy intake (1000-unit increase)	0.70	0.5-1.1		
Average Carbohydrate intake (100-unit increase)	0.9	0.7-1.2		

Average Total fat (100-unit increase)	0.5	0.2-1.3		
Average Protein intake per day (1 unit increase)	0.99	0.98-1.00		
Average fiber intake per day (1 unit increase)	0.99	0.97-1.02		
Average total sugar intake per day (1 unit increase)	0.999	0.993-1.004		
**AST (IU/L) (1 unit increase)**	1.00	0.99-1.02	0.98	0.92-1.03
**ALT (IU/L) (1 unit increase)**	1.01	1.00-1.02	1.03	0.98-1.07
**Ferritin (ng/mL) (100-unit increase)**	1.05	0.95-1.16		
**DEXA**				
Total Fat (g,100-unit increase)	1.02	1.0-1.03		
Total percent fat (%,1 unit increase)	1.08	1.01-1.16		
Trunk fat (g, 100-unit increase) [*]	1.06	1.02-1.09	1.07	1.04-1.11
**Vitamin E (mg) (1 unit increase)**	0.93	0.86-1.02		
**HOMA score**	1.43	1.02-2.01		

[*] Final model adjusted without metabolic syndrome

[a] Final model including age, sex, race, household income, physical activity, smoking status, ALT, AST and with either metabolic syndrome or metabolic syndrome components (Trunk fat, elevated triglycerides, low HDL cholesterol, elevated blood pressure and elevated fasting glucose), using backward elimination methods, with stay p<0.15.

Table S4. Multivariable analysis for factors associated with non-obese NAFLD**,** restricting non-obese Asian on BMI<23kg/m^2

**Variables**	**Crude OR**	**95%CI**	**Multivariable adjusted OR**[a]	**95%CI**
**Age**				
1 unit increase				
20-29	Ref		Ref	
30-39	4.0	1.2-13.5	3.1	0.9-11.2
40-49	4.0	0.9-17.8	3.0	0.5-17.1
50-59	9.6	2.3-39.3	5.6	1.2-25.2
60-69	14.1	3.4-57.7	7.5	1.9-30.1
70-79	15.3	3.6-66.2	7.5	1.9-30.1
80-89	14.7	2.8-76.0	4.0	0.6-29.0
**Sex**				
Male	1.5	0.8-2.9	2.4	0.9-6.1
Female	Ref		Ref	
**Race**				
Non-Hispanic White	Ref		Ref	
Non-Hispanic Black	0.6	0.2-2.4	0.7	0.2-1.8
Hispanics	0.7	0.3-1.4	0.6	0.3-1.4
Asian Americans	1.4	0.6-3.0	1.3	0.5-3.2
Other	0.5	0.1-2.4	0.8	0.3-2.4
**Household income**				
<$55,000	Ref		Ref	
≥$55,000	0.99	0.44-2.20	0.8	0.4-1.9
**Acculturation**				
Born in the U.S.	Ref			
<20 years in the U.S.	2.0	0.5-8.3		
>=20 years in the U.S.	3.6	1.2-11.0		
**Marital status**				
Never married	Ref			
Married or living with partner	7.5	2.6-21.5		
Windowed, divorced or separated	7.6	2.1-26.9		
**BMI (1 unit increase)**	1.6	1.3-1.9		
**Metabolic Syndrome**	8.8	3.7-20.5	7.5	3.1-18.3
**Elevated waist circumference**	2.7	0.97-7.63	2.2	0.9-5.8
**Elevated triglycerides**[*]	5.6	2.0-15.9	4.0	1.8-9.1
**Low HDL cholesterol**[*]	2.6	1.0-6.4	1.8	0.9-3.4
**Elevated blood pressure**[*]	2.1	0.8-5.1	1.0	0.4-2.3
**Elevated fasting glucose**[*]	10.8	5.2-22.6	4.4	2.1-9.4
**Self-reported CVD**	4.8	1.2-20.0		
**Smoking**				
Nonsmoker	Ref		Ref	
Former smoker	2.1	0.2-18.5	1.7	0.1-30.4
Current smoker	0.2	0.1-0.9	0.2	0.1-0.7
**Alcohol drinking**				
Never	Ref			
Light to Moderate	1.2	0.6-2.4		
Heavy	0.3	0.1-0.8		
**Physical activity**				
Inadequate	3.7	1.5-9.1		
Adequate	Ref		3.6	1.3-9.8
**Macronutrients**			Ref	
Average total energy intake (1000-unit increase)	0.7	0.4-1.1		
Average Carbohydrate intake (100-unit increase)	0.8	0.6-1.2		
Average Total fat (100-unit increase)	0.4	0.1-1.3		
Average Protein intake per day (1 unit increase)	0.99	0.97-1.00		

Average fiber intake per day (1 unit increase)	0.99	0.96-1.01		
Average total sugar intake per day (1 unit increase)	0.999	0.993-1.004		
**AST (IU/L) (1 unit increase)**	1.00	0.99-1.01	0.99	0.93-1.05
**ALT (IU/L) (1 unit increase)**	1.01	0.996-1.024	1.01	0.96-1.06
**Ferritin (ng/mL) (100-unit increase)**	1.04	0.93-1.16		
**DEXA**				
Total Fat (g,100-unit increase)	1.02	1.01-1.04		
Total percent fat (%,1 unit increase)	1.08	1.01-1.17		
Trunk fat (g, 100-unit increase) [*]	1.06	1.02-1.10		
**Vitamin E (mg) (1 unit increase)**	0.93	0.85-1.02		
**HOMA score**	1,55	1.11-2.17		

[*] Final model adjusted without metabolic syndrome
[a] Final model including age, sex, race, household income, physical activity, smoking status, ALT, AST and with either metabolic syndrome or metabolic syndrome components (elevated waist circumference, elevated triglycerides, low HDL cholesterol, elevated blood pressure and elevated fasting glucose), using backward elimination methods, with stay p<0.15.
